# Characterization of pain-related behaviors and gene expression profiling of peripheral sensory ganglia in a mouse model of acute ankle sprain

**DOI:** 10.3389/fnbeh.2023.1189489

**Published:** 2023-05-25

**Authors:** Yushuang Pan, Qimiao Hu, Yunqin Yang, Huimin Nie, Chengyu Yin, Huina Wei, Yan Tai, Boyu Liu, Zui Shen, Xiaofen He, Jianqiao Fang, Boyi Liu

**Affiliations:** ^1^Key Laboratory of Acupuncture and Neurology of Zhejiang Province, Department of Neurobiology and Acupuncture Research, The Third Clinical Medical College, Zhejiang Chinese Medical University, Hangzhou, China; ^2^Academy of Chinese Medical Sciences, Zhejiang Chinese Medical University, Hangzhou, China

**Keywords:** ankle sprain, pain, RNA-Seq, aversion, dorsal root ganglia, spinal cord

## Abstract

**Introduction:**

Lateral ankle sprain (LAS) is a very common type of joint injury. It occurred with high incidence among general population and especially among individuals participating sports and outdoor activities. A certain proportion of individuals who once developed LAS may suffer persistent ankle pain that affects daily activities. However, the mechanisms underlying LAS-induced pain still remained largely unknown.

**Methods:**

We established a LAS mouse model and systematically evaluated the pain-related behaviors in this mouse model. RNA sequencing (RNA-Seq), combined with bioinformatics analysis, was undertaken to explore gene expression profiles. Immunostaining was used to study glial cell and neuron activation in ipsilateral spinal cord dorsal horn (SCDH) of LAS model mice. Ibuprofen was used to treat LAS model mice.

**Results:**

The LAS model mice developed obvious signs of mechanical and heat hypersensitivities as well as gait impairments in ipsilateral hind paws. Besides, LAS model mice developed signs of pain-related emotional disorder, including pain-induced aversion. By RNA-Seq, we were able to identify certain differentially expressed genes and signaling pathways that might contribute to pain mechanisms of LAS mouse model. In addition, LAS model mice showed increased c-Fos and p-ERK immunoreactivity as well as astrocyte and microglia overactivation in ipsilateral spinal cord dorsal horn, indicating central sensitization might occur. Finally, LAS model mice respond to ibuprofen, a drug clinically used to treat ankle sprain pain.

**Conclusion:**

Our study found LAS model mice may be used as a preclinical animal model for screening novel targets or therapies for ankle sprain. Thus, the study may further help to understand molecular mechanisms contributing to ankle sprain-induced pain.

## Introduction

Joint injuries usually result in persistent joint pain and locomotion impairments (Alghadir et al., 2020). Ankle sprain is one of the most common types of joint injuries that impose heavy financial burdens on the healthcare system (Ivins, 2006; Bielska et al., 2019). A majority of ankle sprains are lateral ankle sprains (LASs), which are caused by the disruption of the lateral ligament complex due to excessive plantar flexion and foot inversion (Garrick and Requa, 1988; Vuurberg et al., 2018). It occurred with high incidence among people, especially in individuals participating in sports and outdoor activities (Vuurberg et al., 2018; Michels et al., 2022; Tee et al., 2022). Unfortunately, a certain proportion of individuals who once developed LAS may suffer persistent ankle pain and recurrent sprains (Anandacoomarasamy and Barnsley, 2005; de Ruvo et al., 2022; Michels et al., 2022). The recurrent sprain and associated ankle pain may lead to significant disturbance in daily activities and even cause psychological impairments, including injury-related fear, sadness, and anxiety among the suffering individuals (Kosik et al., 2020; Bain et al., 2022).

Although ankle sprain is a highly prevalent joint injury, the mechanisms underlying ankle sprain-induced pain still remained largely unknown. Currently, most of our understanding of ankle sprain-induced pain comes from clinical observations or data derived from some other types of joint disorders, such as joint inflammation. Therefore, understanding the mechanisms underlying ankle sprain-induced pain may help to identify targeted therapies to manage ankle sprain.

Transecting of anterior talofibular ligament (ATFL) and calcaneal fibular ligament (CFL), two lateral ligaments of the mouse ankle, is an established animal model to mimic LAS (Hubbard-Turner et al., 2013). The LAS model mice showed reduced physical activities, impaired balance, and gait, mimicking results observed from individuals who had acute LAS (Hubbard-Turner et al., 2013). Although this animal model was developed to mimic human ankle sprain, it still remains unknown whether the LAS model mice develop any pain-related behavior. Therefore, we planned to systematically characterize the detailed pain-related behaviors in this mouse model. We further performed RNA-Seq to profile gene expression changes in ipsilateral dorsal root ganglion (DRG) of LAS model mice, aiming to explore potential genes or signaling pathways involved in ankle sprain-induced pain.

## Methods and materials

### Animals

All animal care and experimental procedures were approved by the Laboratory Animal Management and Welfare Ethical Review Committee of Zhejiang Chinese Medical University (Permission number: IACUC-20190819-04). C57BL/6 mice (6–8 weeks of age, male and female) were used in this study. All animals were housed in facilities accredited by the Association for Assessment and Accreditation of Laboratory Animal Care (AAALAC) under standard environmental conditions (12 h light–dark cycles and 23°C).

### LAS model establishment

**Figure 1:**
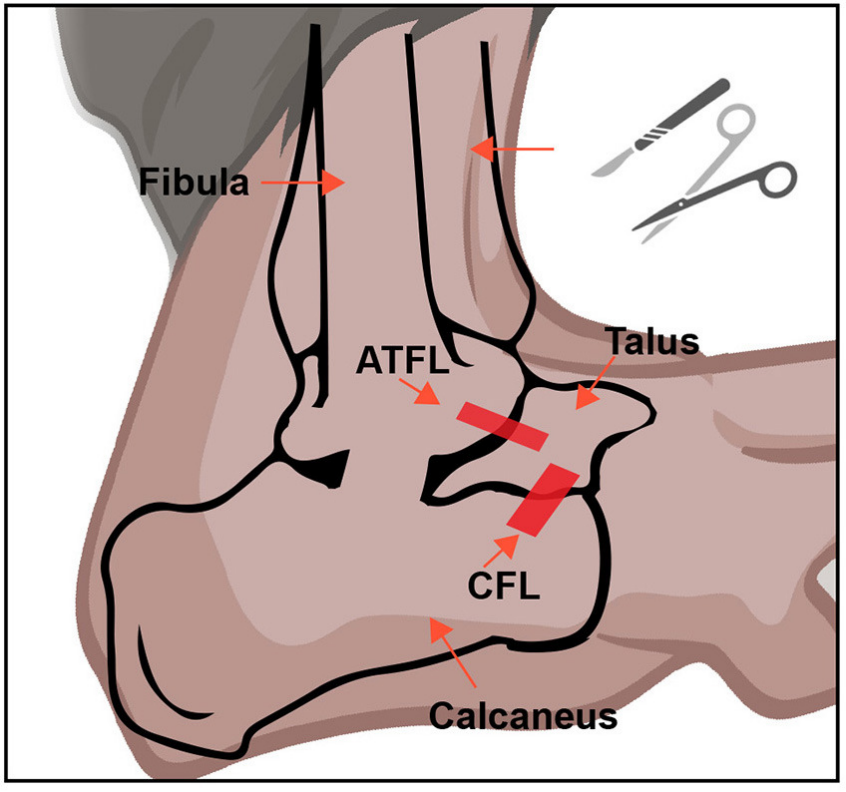
Establishment of the LAS mouse model. A schematic picture of ATFL and CFL in the mouse ankle joint. This figure was created with BioRender.com.

The mouse was anesthetized using isoflurane (4%). The right ankle of each mouse was then shaved and disinfected using iodophor beforehand. The major procedures were adopted from a previous study (Hubbard-Turner et al., 2013). Briefly, for the LAS group, a small incision in the ankle was made, and the ATFL and CFL were both transected, and the skin was then closed with a 6-0 surgical suture. For the sham group, a small incision was retracted in the same place as the LAS group, but no ligament was cut. After the surgery was completed, the mouse was transferred to a heating pad maintained at 37°C and allowed to recover. The anatomical position of the transection site is shown in Figure 1.

### Paw withdrawal threshold (PWT) test

Detailed procedures were described in our previous publication (Chai et al., 2018; Yin et al., 2020a). Briefly, the mice were individually placed in transparent Plexiglas chambers on an elevated mesh floor and were habituated for 30 min before the test. The mechanical allodynia was examined with a series of von Frey filaments (from 0.04 to 4 g) applied in proper order to the heel area of the hind paw. The minimum force that caused the mouse to withdraw its hind paw away from the filament was considered the withdrawal threshold. For each mouse, a von Frey hair was applied at 5–10 s intervals. The threshold was determined using the “Up–Down” testing paradigm, and the 50% PWT was calculated by the non-parametric Dixon test (Dixon, 1980; Chaplan et al., 1994).

### Paw withdrawal frequency (PWF) test

Paw withdrawal frequency was measured by applying the 0.07 g or 0.4 g von Frey filament to the heel area of the hind paw a total of 10 times. The PWF was derived by dividing the number of paw withdrawals by the 10 applications and recording it as %.

### Hargreaves test

The plantar test apparatus (Ugo Basile, Italy) was used to evaluate thermal hyperalgesia according to the methods we previously described (Hu et al., 2019). A radiant light beam generated by a light bulb was directed into the right hind paw to determine the paw withdrawal latency (PWL). A 20 s cutoff threshold was set to avoid excessive heating that can cause tissue damage. A significant decrease in PWL was interpreted as heat pain hypersensitivity.

### Acetone test

Mice were placed in transparent Plexiglas chambers on an elevated mesh floor and calmed down 30 min before the test. Acetone (20 μl) was dabbed or sprayed on the plantar surface of the heel area of the ipsilateral hind paw of each mouse as previously described (Liu et al., 2013). The licking and shaking of the affected hind paw in response to the acetone application were recorded with a camera. The duration of the licking or shaking of the hind paw was analyzed thereafter.

### Gait analysis

The Digi gait analysis system (Mouse Specifics, Inc., USA) was employed to evaluate the gait pattern of mice. The mice walked through an illuminated glass platform with a treadmill, while a video camera recorded the behavior from below. The belt speed was set at 18 cm/s constant speed. Each mouse was trained for a consecutive 3 days before the formal test. Gait parameters were then analyzed thereafter.

### Balance beam test

The beam was a 1 m-long round piece of wood with a 20 mm diameter that was elevated above the bench surface and at a 15-degree angle to the ground. A light bulb was placed on one side to force each mouse to move. On the other side, it was connected to a dark box (20 × 20 × 20 cm) for the mouse to land safely. Each mouse was allowed to be trained for 3 days, placed at the start of the beam, and allowing them to cross the beam to the dark box. The duration to cross the beam of mice was recorded.

### Real-time PEA test

The real-time PEA chambers were put on the mesh floor. It contained two chambers. The size of each chamber was 50 × 28 × 32 cm, made with plastic plates that had cross stripes or vertical stripes to distinguish them from each other. For the first 10 min, the mouse was allowed to explore both chambers without any mechanical stimulation (pre-phase). After the pre-period, the mouse usually showed a small preference for one of the two chambers. For the second 10 min, 0.4 g von Frey hair was used to simulate the hind paw whenever the mouse entered the preferred chamber. For the last 10 min, the mouse was allowed to freely explore both chambers (post-phase). The movements of each mouse in each chamber were recorded via Any-Maze recording system (Stoelting, USA) and analyzed thereafter.

### Tissue collection and RNA extraction

The whole procedure was derived from our previous studies (Yin et al., 2019; Nie et al., 2021). Briefly, the L3–5 DRG segments ipsilateral to the side of the ankle sprain injury were harvested and used for generating libraries for RNA-Seq. On day 14, mice were anesthetized with sodium pentobarbital and were perfused with saline. After perfusion, the L3–5 DRG segments were immediately preserved in an RNAlater solution (Thermo Fisher Scientific, USA). Total RNA was extracted using TRIzol reagent (Thermo Fisher Scientific, USA) according to the manufacturer's instructions with DNaseI to degrade contaminating DNA. The purity and concentration of the samples were assessed by the NanoDrop Spectrophotometer (NanoDrop Products, CA, USA), and the RNA integrity was assessed by the Agilent 2100 Bioanalyzer (Agilent Technologies, Palo Alto, CA).

### RNA-Seq and bioinformatics analysis

The samples were sequenced by BGISEQ-500 from BGI Group (Shenzhen, China). Raw sequencing reads were aligned to the mouse genome. Differential expression analyses were performed with R and Bioconductor packages of edgeR and limmavoom as reported in our previous studies (Chen et al., 2020; Xu et al., 2022). The threshold required for the genes to be considered significantly changed was as follows: *q*-value ≤0.05 and the absolute value of |log_2_ (fold change)| ≥ 1.5.

### Functional enrichment analysis of DEGs

Functional enrichment analysis was performed through the functional annotation package the “cluster Profiler” in R studio software (RStudio, Boston, MA, USA). GO analysis was also conducted. For each enriched function term, the Q-value of enriched functions and the *Q*-value by multiple testing corrections were calculated by the “cluster Profiler” package in R studio software. The GO functional enrichment analysis was performed for DEGs using the Database for Annotation, Visualization, and Integrated Discovery (DAVID) online tools (http://www.geneontology.org).

### Protein–protein interaction (PPI) network analysis

The search instrument for the retrieval of interacting genes (STRING) was adopted to provide information regarding predicted and experimental interactions of proteins, and the prediction method of this database is from the neighborhood, gene fusion, co-occurrence, co-expression experiments, databases, and text mining. By setting the Combination score >0.4 as the reliability threshold value, the web-based STRING database (http://string-db.org/) was used to produce PPI predictions after uploading the union gene list to the search bar. Based on the relationships, a PPI network was established and then displayed using the Cytoscape software. The consistency degree between proteins, namely the number of proteins it connected, was calculated to weigh its significance in this network.

### Immunofluorescence staining

Mice were deeply anesthetized with sodium pentobarbital and were perfused with 4°C saline and then with 4% paraformaldehyde transcardially as previously described (Liu et al., 2022). The spinal cord specimens were harvested and then transferred to 15% sucrose for 24 h and 30% sucrose for dehydration. Tissues were serially cut into 14 mm thick sections on a frozen microtome (CryoStar NX50, Thermo Fisher, CA, USA) and mounted on gelatin-coated glass slides as six sets of every fifth serial section. All the slides were blocked with 5% normal donkey serum in PBS (with 0.3% Tween-20) for 1 h at 37°C and then incubated overnight with corresponding primary antibodies. The primary antibodies used were mouse anti GFAP (1:500, #c9205, Sigma), rabbit anti c-Fos (1:500, #2250, CST), rabbit anti p-ERK (1:500, #4370, CST), and rabbit anti CD68 (1:500, #NB100-683PE, Novus). The next day, the sections were rinsed with PBS (6 × 10 min) and incubated for 1 h with a mixture of corresponding secondary antibodies. Fluorescence images were captured by a Nikon A1R laser scanning confocal microscope (Nikon, Japan). All stained sections were examined and analyzed in a blinded manner. Three images were randomly selected per mouse tissue and averaged and then compared as described in our previous studies (Liu et al., 2016; Zhang Y. et al., 2022).

### Drug administration

Ibuprofen (#NSC256857, Selleck) was dissolved in DMSO and then diluted with 0.9% sterile saline to the required volume and applied at a concentration of 30 mg/kg. Ibuprofen and vehicle were injected i.p. on days from 8 to 14. The ibuprofen drug dosage we chose was based on previous reports (Caceres et al., 2017).

### Statistical analysis

All data in the article are expressed as the mean ± SEM. An unpaired *t*-test was used for comparisons between the two groups. One-way ANOVA or two-way ANOVA followed by Bonferroni's *post-hoc* test was used to compare data among three or more groups. A *p*-value of <0.05 was considered statistically significant. Statistical analyses were performed using GraphPad Prism 9.0 software (GraphPad Software Inc., CA, USA).

## Results

### LAS model mice showed gait abnormality and balance disorder

According to a previous study (Hubbard-Turner et al., 2013), we established the mouse model for lateral ankle sprain (LAS) by transecting both ATFL and CFL as shown in Figure 1. Ankle sprain patients usually exhibit impairments in gait that can affect daily activities (Punt et al., 2015). We then began to examine whether the LAS model mice exhibit similar gait abnormalities. The gait behaviors of sham and LAS model groups were recorded using DigiGait analyzing system 14 days after model establishment and analyzed thereafter (Supplementary Figure 1A). Gait analysis revealed that LAS model mice showed impairment in gait behavior 14 days after model establishment vs. sham group (Supplementary Figures 1A–D). Some specific gait parameters, which include averaged paw area ration, maximal paw area ratio, stance ratio, and stride length ratio were all significantly decreased in LAS model mice compared with sham group mice (Supplementary Figures 1E–G).

Next, the balance beam test was applied to assess the balance and motor capabilities of mice 14 days after the model establishment (Supplementary Figure 1I). When compared with the sham group, LAS model group mice spent significantly more time passing the inclined beam as shown in Supplementary Figure 1J. These results demonstrate that LAS model mice showed obvious deficits in gait behavior as well as balance and motor capability, a result consistent with patients suffering from ankle sprain.

### LAS model mice developed sustained mechanical and heat pain hypersensitivities in affected ankles

A prominent feature of ankle sprain patients is the development of ankle pain that affects their life quality (Gonzalez de Vega et al., 2013; Gaddi et al., 2022). Although the LAS model has been developed to mimic human ankle sprain, it still remains unknown whether the LAS model mice develop similar ankle joint pain as human patients. Therefore, we aimed to explore the potential pain responses of LAS model mice with a series of pain behavioral assays.

**Figure 2:**
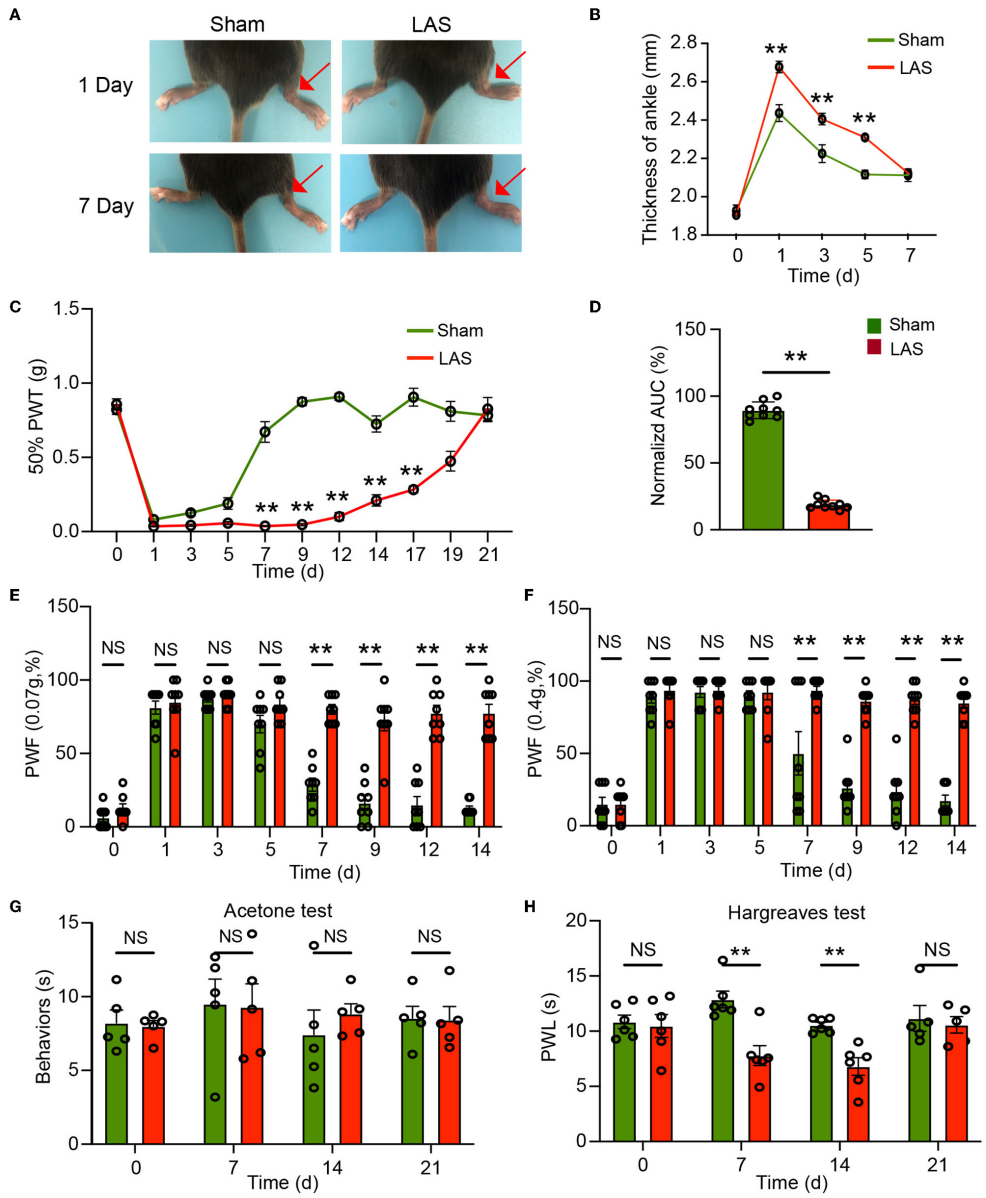
Pain-related behavior evaluations of male LAS model mice. **(A)** Representative photos showing the ipsilateral ankles (denoted with red arrow) of male sham and LAS model mice 1 and 7 days after the operation. **(B)** Time course showing the changes in ankle thickness of male sham and LAS model mice from days 0 to 7. **(C)** Time course showing the changes in 50% paw withdrawal threshold (PWT) of the ipsilateral hind paw of male sham and LAS model mice from days 0 to 21. **(D)** Area under the curve (AUC) analysis of corresponding traces in **(C)**. **(E, F)** Paw withdrawal frequency (PWF) of the ipsilateral hind paw of male sham and LAS model mice in response to specific mechanical stimuli (0.07 or 0.4 g, respectively). **(G)** Cold pain hypersensitivity evaluation of ipsilateral hind paw by acetone test of male sham and LAS model mice. **(H)** Heat pain hypersensitivity evaluation of ipsilateral hind paw by Hargreaves test of male sham and LAS model mice. *n* = 5–9 mice/group. **p < 0.01 vs. sham group. Student's *t*-test was used for statistics in **(D)**. Two-way ANOVA followed by Bonferroni's *post-hoc* test was used for statistics in all other panels.

After transecting the ATFL and CFL ligaments, the ipsilateral ankle joint of LAS model mice began to show robust swelling 1 day later compared with sham group mice (Figure 2A). The ankle swelling gradually returned to normal after 7 days (Figure 2B). We started by testing the mechanical hypersensitivities using von Frey hair test. Before model establishment, the two groups of mice exhibit similar 50% PWT in the ipsilateral hind paw (day 0, Figure 2C). After the model was established, although both groups of mice showed robust mechanical pain (with no statistical difference from days 1 to 5, Figure 2C), 50% PWT gradually recovered in sham group mice and almost completely recovered 7 days later after sham surgery. In contrast, the LAS model mice developed persistent mechanical pain that lasts over 2 weeks compared with the sham group (Figure 2C). Area under the curve analysis from days 5 to 19 indicated an accumulated reduction of 50% PWT in the LAS model mice compared with sham group mice (Figure 2D). We further studied the paw withdrawal frequency (PWF) with a specific von Frey hair (0.07 or 0.4 g) in sham and LAS model mice. As shown in Figures 2E, F, LAS model mice exhibited significantly higher PWF in responses to 0.07 or 0.4 g challenge from days 7 to 14 compared with sham group mice.

We next tested thermal pain in LAS model mice. Cold pain hypersensitivities were evaluated by the acetone test. As shown in Figure 2G, the two groups of mice did not exhibit any difference in cold pain hypersensitivity in response to the acetone challenge. We continued to examine the heat pain hypersensitivities. Hargreaves test revealed that LAS model mice developed obvious heat pain hypersensitivity in response to noxious heat stimulation starting from days 7 to 14, compared with sham group mice (Figure 2H). Therefore, these results demonstrate that LAS model mice exhibit mechanical and heat pain hypersensitivities.

**Figure 3:**
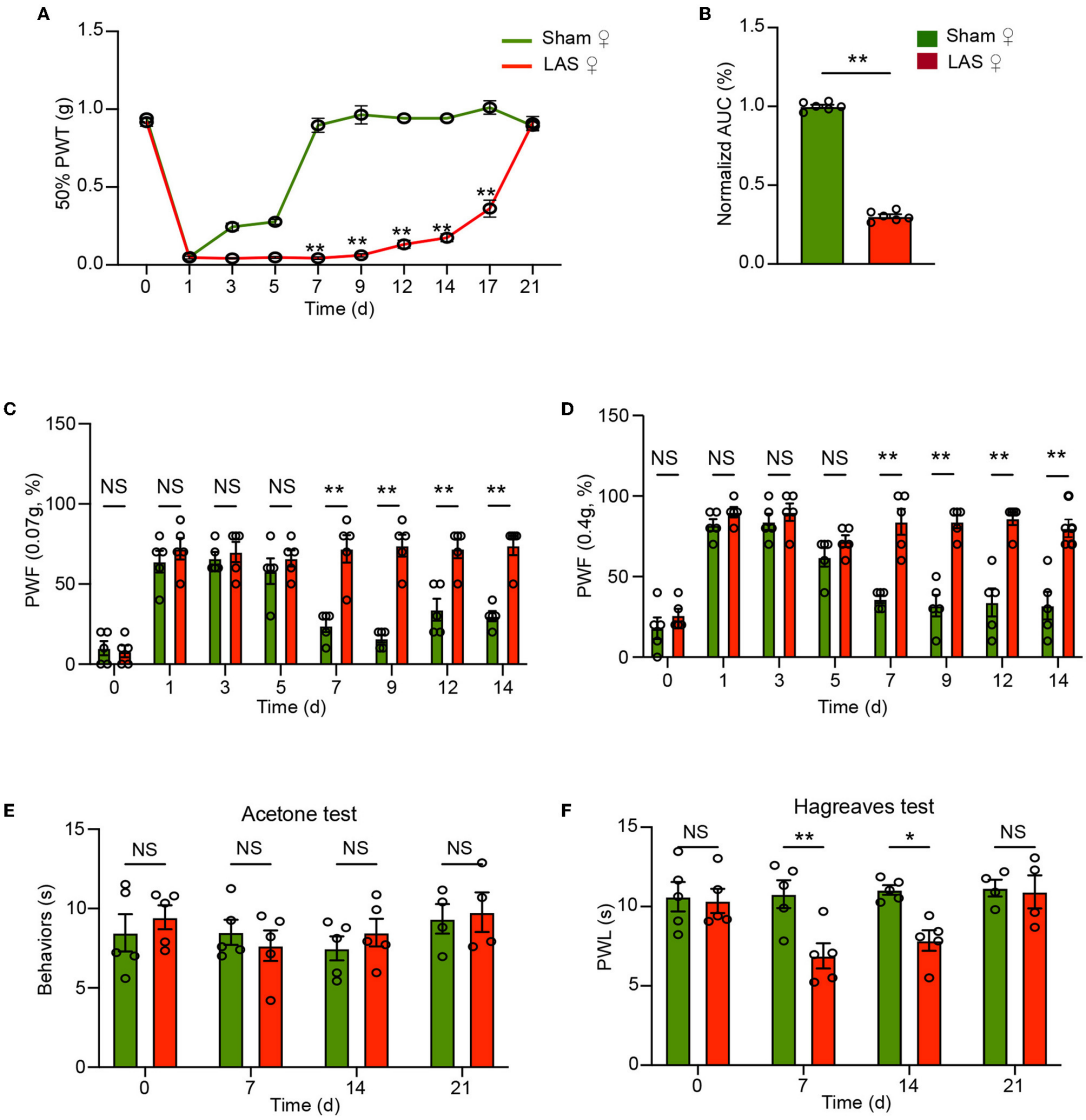
Pain-related behavior evaluations of female LAS model mice. **(A)** Time course showing the changes in 50% paw withdrawal threshold (PWT) of the ipsilateral hind paw of female sham and LAS model mice from days 0 to 21. **(B)** Area under the curve (AUC) analysis of corresponding traces in **(A)**. **(C, D)** Paw withdrawal frequency (PWF) of the ipsilateral hind paw of female sham and LAS model mice in response to specific mechanical stimuli (0.07 or 0.4 g, respectively). **(E)** Cold pain hypersensitivity evaluation of ipsilateral hind paw by acetone test of female sham and LAS model mice. **(F)** Heat pain hypersensitivity evaluation of ipsilateral hind paw by Hargreaves test of female sham and LAS model mice. *n* = 5–9 mice/group. **p < 0.01, *p < 0.05 vs. sham group. Student's *t*-test was used for statistics in **(B)**. Two-way ANOVA followed by Bonferroni's *post-hoc* test was used for statistics in all other panels.

The above results were all obtained from male mice. It is known that sex difference plays an important role in pain mechanisms (Sorge et al., 2015; Chen et al., 2018). We then continue to examine the pain response in female LAS model mice. Similar to male mice, female mice also developed obvious mechanical hypersensitivities starting from days 7 to 17, in comparison to the sham group female mice (Figures 3A, B). In addition, female LAS model mice also exhibited significantly higher PWF in response to 0.07 or 0.4 g von Frey hair challenge from days 7 to 14, in comparison to sham group female mice (Figures 3C, D). Female LAS model mice did not exhibit any cold pain hypersensitivity in response to acetone challenge (Figure 3E). Similar to male mice, they showed obvious heat pain hypersensitivity in response to noxious heat stimulation starting from days 7 to day 14, compared with the sham group of female mice (Figure 3F). These results demonstrate that both male and female LAS model mice develop sustained mechanical and heat pain hypersensitivities.

### LAS model mice developed aversive behavior in response to mechanical stimulation

**Figure 4:**
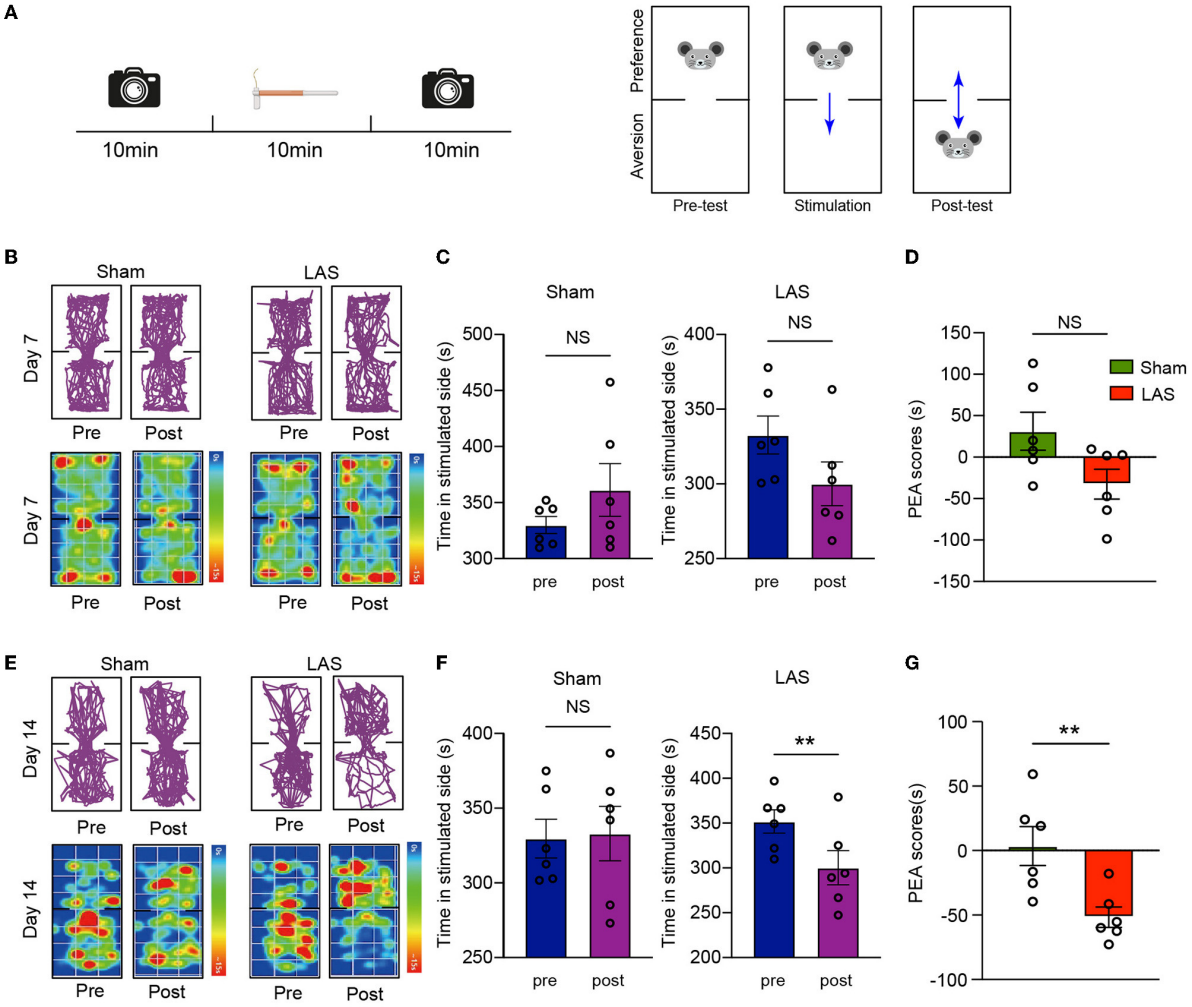
LAS model mice exhibit aversion behavior in real-time place escape/avoidance (PEA) behavior test. **(A)** Cartoon showing real-time PEA test performed on day 7 or day 14 after LAS model establishment. The pre-test phase, stimulation phase, and post-test phase each take 10 min duration as indicated. A von Frey hair of 0.4 g was used to stimulate the hind paw of mice whenever it enters the preferred chamber. The movement of the mouse was videotaped in pre-test and post-test phase and analyzed thereafter. **(B)** Upper panels: representative pictures showing moving traces of mice from sham and LAS model groups during pre- and post-test phases on day 7. Lower panels: corresponding heat map showing the movements of mice. **(C)** Summary of the time spent on the stimulated side during the pre- and post-test phase of sham (left panel) or LAS model (right panel) mice on day 7. **(D)** Summary of PEA scores of sham and LAS model mice on day 7. **(E)** Upper panels: representative pictures showing moving traces of mice from sham and LAS model groups during pre- and post-test phases on day 14. Lower panels: corresponding heat map showing the movements of mice. **(F)** Summary of the time spent on the stimulated side during the pre- and post-test phase of sham (left panel) or LAS model (right panel) mice on day 14. **(G)** Summary of PEA scores of sham and LAS model mice on day 14. **p < 0.01. *n* = 6 mice/group. Student's *t*-test was used for statistics.

Chronic pain may result in emotional disorders, including aversion, anxiety, and depression. Therefore, we aimed to explore whether LAS model mice might develop any signs of emotional disorders. A modified real-time place escape/avoidance (PEA) test using mechanical stimulation was applied to evaluate aversive behavior (Figure 4A). In short, the mouse was put in a two-chamber box that allows free access to explore with no stimulation (pre-stimulation phase, 10 min duration). During the stimulation phase (10 min duration), a repetitive mechanical stimulation by a fixed von Frey hair (0.4 g) was applied to the ipsilateral hind paw of the mice whenever it goes into or dwelled in the preferred chamber. Then the mice freely explored the box without any stimulation (post-stimulation phase, 10 min duration) (Figure 4A). Real-time PEA test revealed that no aversion behavior was observed in LAS model mice 7 days after model establishment (Figures 4B–D). LAS group mice spent significantly less time in a mechanically stimulated chamber and exhibited apparent aversion behavior during the post-stimulation phase vs. sham group mice 14 days after model establishment (Figures 4E–G). This result indicates that LAS model mice exhibit mechanical pain-induced aversive behavior.

### RNA-Seq analysis of ipsilateral DRG tissues from the LAS mouse model

**Figure 5:**
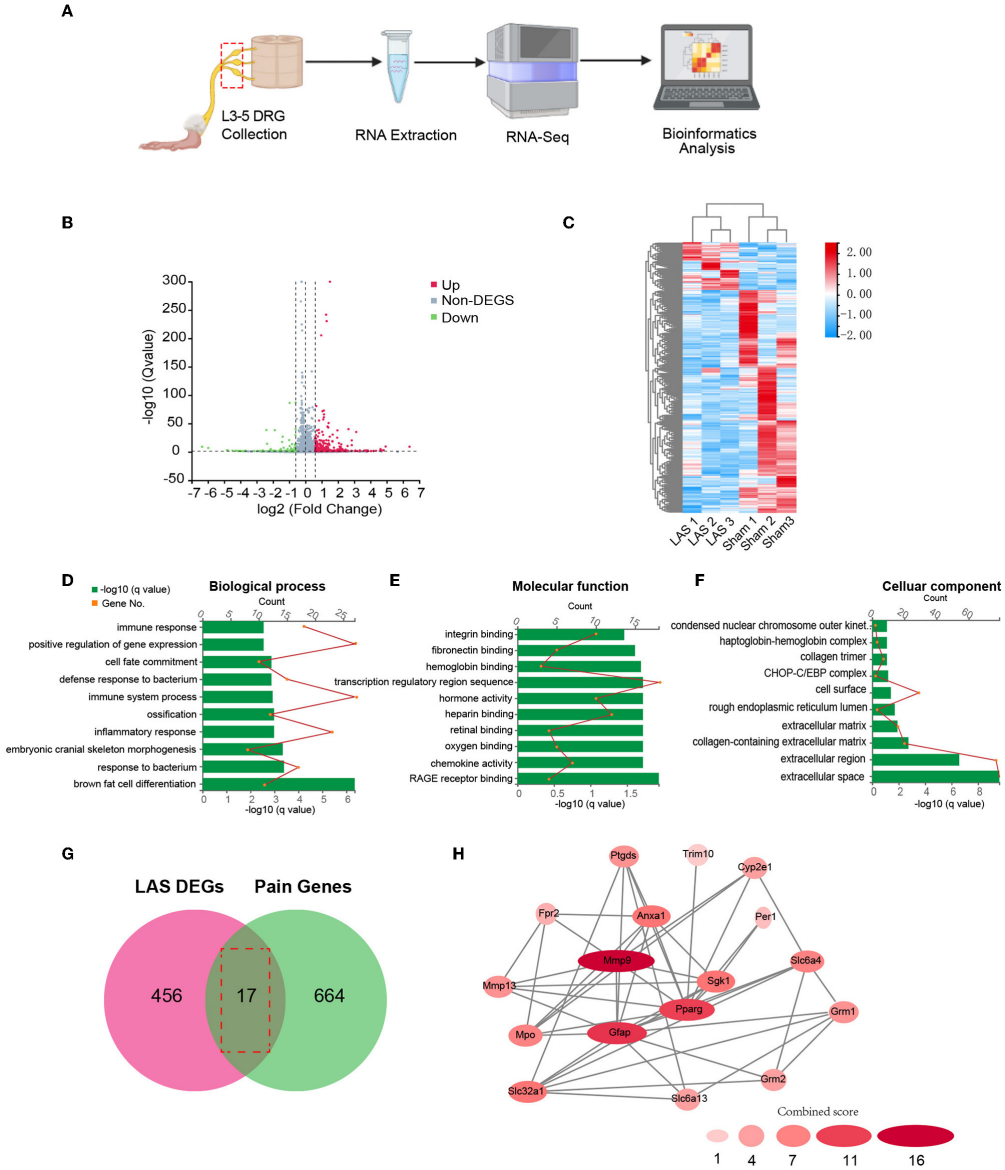
Gene expression profiling of ipsilateral dorsal root ganglia of sham and LAS model mice using RNA-Seq. **(A)** Flow chart showing ipsilateral L3–L5 DRG collection, RNA extraction, RNA-Seq, and bioinformatics analysis of this study. **(B)** Volcano plot illustrates gene expressions of the LAS model group vs. Sham group. Red and green dots represent up and downregulated DEGs, respectively. Non-DEGs were indicated by gray color. **(C)** Hierarchical clustering of overall gene expression of the LAS model group vs. Sham group shown by heat map. **(D–F)** GO pathway analysis of all DEGs. The top 10 significantly enriched biological processes **(D)**, molecular functions **(E)**, and cellular components **(F)** of all DEGs were listed. **(G)** Overlapping of DEGs with pain-related genes. **(H)** PPI network analysis of DEGs overlapped with pain-related genes.

We next performed gene expression profiling of DRG from LAS model mice vs. sham group mice by RNA-Seq on day 14. Ipsilateral L3–L5 DRG that innervates the affected hind limb was collected for RNA-Seq and bioinformatics analysis. Figure 5A indicates the overall workflow chart of these experiments. Each sample generated ~23.92 M (million) raw reads. The clean reads ratio for each sample was above 97.0%. Approximately 94% of total reads were successfully matched to the mouse genome, and a total of 22,064 genes were identified by the sequencing (Table 1). We then screened the differentially expressed genes (DEGs) by the criteria of fold change ≥1.5 or ≤-1.5, with an FDR *q*-value of ≤0.05. In total, we found 473 DEGs, which include 389 up and 84 downregulated genes. The top 15 up and downregulated DEGs are further illustrated in Tables 2, 3, respectively. All these DEGs were further illustrated by volcano plot and heat map as shown in Figures 5B, C. To better understand the molecular mechanisms underlying the pain of the LAS model mice, we performed gene ontology (GO) analysis. The results of the GO analysis are shown in Figures 5D–F. As can be seen, the most enriched biological processes include immune response and positive regulation of gene expression (Figure 5D). The mostly enriched molecular function included transcription regulatory region sequence and integrin binding (Figure 5E). The most enriched cellular components among total DEGs included extracellular space and extracellular region (Figure 5F). We further aimed to explore the genes related to pain processing in DRG from the LAS model mice. We retrieved the dataset for pain-related genes and compared it with the DEGs we identified from our RNA-Seq (Chung et al., 2016). In this way, we identified 17 DEGs that are potentially related to pain mechanisms (Figure 5G). Protein–protein interaction (PPI) analysis was further performed on these 17 genes, and the major hub identified from the network included *Mmp9, Pparg*, and *Gfap* (Figure 5H). These data suggest that pain-related genes were identified from the DRG of LAS model mice. These genes may contribute to ankle sprain-induced pain.

**Table 1:** Information on total reads and mapping ratio for Sham and LAS groups in RNA-Seq.

**Sample**	**Total raw reads (M)**	**Total clean reads (M)**	**Total clean bases (Gb)**	**Clean reads Q20 (%)**	**Clean reads Q30 (%)**	**Clean reads ratio (%)**
LASDRG1	23.92	23.81	1.19	97.32	92.87	99.52
LASDRG2	23.92	23.78	1.19	97.26	92.68	99.38
LASDRG3	23.92	23.78	1.19	97.32	92.83	99.39
ShamDRG1	23.92	23.82	1.19	97.41	93.12	99.55
ShamDRG2	23.92	23.83	1.19	97.27	92.77	99.61
ShamDRG3	23.92	23.82	1.19	97.22	92.64	99.56

**Table 2:** The Detailed Information of the top 15 up-regulated DEGs.

**Upregulated gene**	**Gene ID**	**Log2 fold change**	***Q*-value**	**Official gene name**
*Krt5*	110308	6.397402978	5.86E-10	Keratin 5
*Gm6943*	629055	4.928416993	2.77E-10	Predictedgene, 6943
*Gm37013*	100384868	4.805140956	0.00116316	Predictedgene, 37013
*Gm40378*	105244844	4.803048577	0.001175299	Predictedgene, 40378
*Gm38667*	103611159	4.741010619	0.001610817	Predictedgene, 38667
Gm10408	100041840	4.617862388	0.002866511	Predicted gene, 10408
*Krt84*	16680	4.612495878	8.02E-06	Keratin 84
*Edar*	13608	4.603507095	0.003057919	Ectodysplasina receptor
*Alx3*	11694	4.340472689	0.008991439	Arista less-like homeobox 3
*DXBay18*	574405	4.188469596	0.015475723	PWWP domain containing 4B
*Dcstamp*	75766	4.188469596	0.015475723	Dendrocyte expressed seven transmembrane protein
*Mup10*	100039008	4.038246509	0.025081434	Major urinary protein 10
*Hoxc13*	15422	4.018544594	0.026533848	Homeobox C13
*Trpc2*	22064	3.838212778	0.044169452	Transient receptor potential cation channel, subfamily C, member 2
*Gata6*	14465	3.825899516	0.002228361	GATA binding protein 6

**Table 3:** The detailed information of the top 15 down-regulated DEGs.

**Downregulated gene**	**Gene ID**	**Log2 fold change**	**Q value**	**Official gene name**
*Ttc30a2*	620631	−6.361353569	7.43E-10	Tetratricopeptide repeat domain 30A2
*Gm46340*	108167989	−6.359967029	7.56E-10	Predicted gene, 46340
*Htd2*	109729085	−5.982807299	9.19E-08	Hydroxyacyl-thioester dehydratase HTD2
*Gm52417*	115489097	−4.833454243	9.10E-04	Predicted gene, 52417
*Gm42427*	115490184	−4.7774301	0.001228705	Predicted gene, 42427
*Gm21104*	100861647	−4.491151248	0.004645249	Predicted gene, 21104
*Xlr3c*	22446	−4.297601148	0.009932256	X-linked lymphocyte-regulated 3C
*Gm33933*	102637020	−4.198686122	0.014165461	Predicted gene, 33933
*Gm906*	380882	−4.127132861	0.018029292	Predicted gene, 906
*LOC101055672*	101055672	−4.038038934	0.024019665	Nuclear body protein SP140-like
*Gm8220*	666660	−3.997595108	0.026993597	Predicted gene 8220
*Gm45929*	105980076	−3.876758027	0.038265773	Myocyte enhancer factor 2B like
*Gm6749*	105242736	−3.84341077	0.041981288	Predicted pseudogene 6749
*Bcl2a1d*	12047	−3.788810328	0.048322007	B cell leukemia/lymphoma 2 related protein A1d
*Lrit3*	242235	−3.681895124	0.004278925	Leucine-rich repeat, immunoglobulin-like and transmembrane domains 3

### LAS model mice showed increased c-Fos and p-ERK immunoreactivity as well as glial cell overactivation in the ipsilateral spinal cord dorsal horn

**Figure 6:**
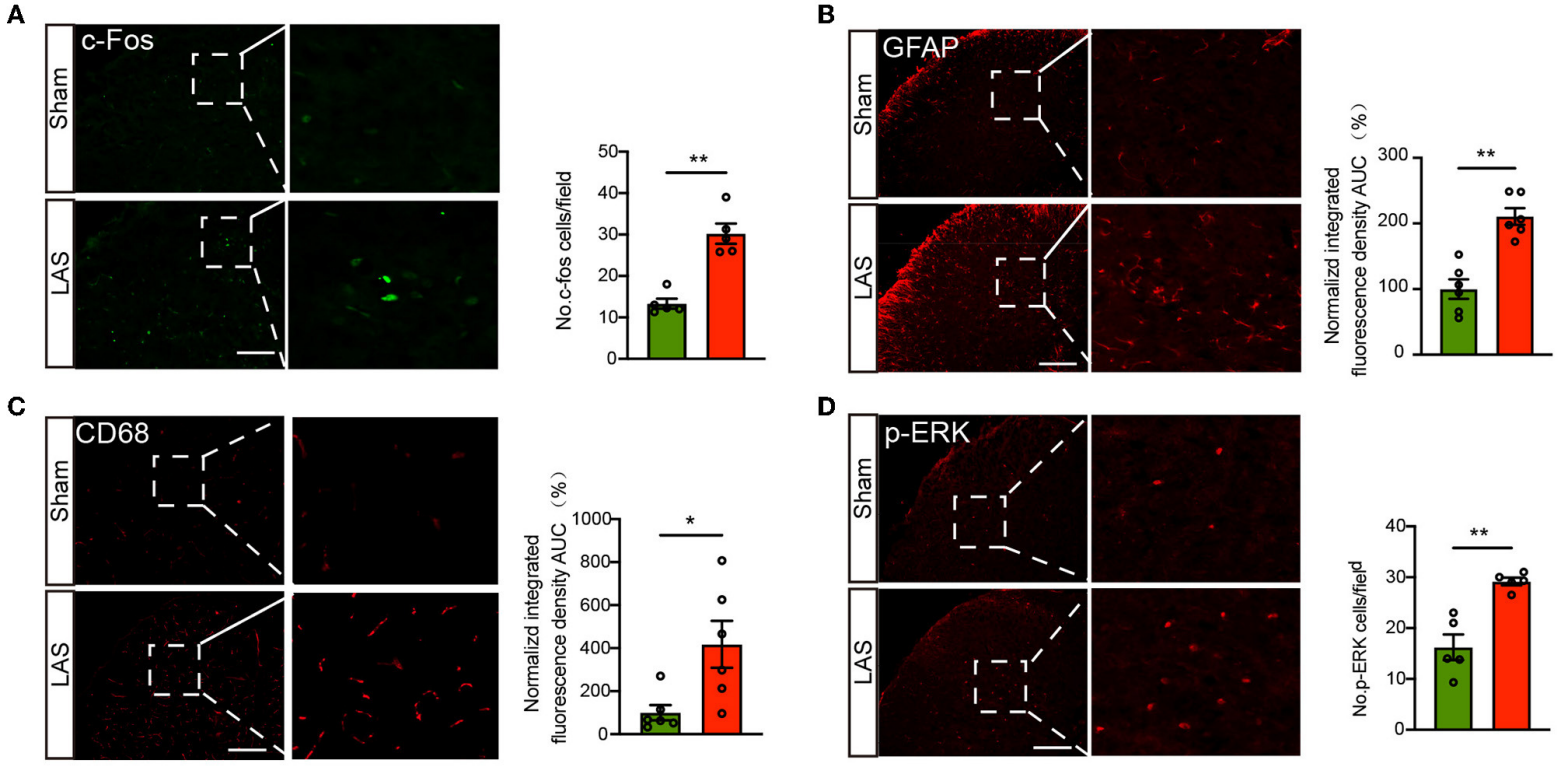
LAS model mice showed increased c-Fos and p-ERK immunoreactivity as well as glial cell overactivation in the ipsilateral spinal cord dorsal horn. **(A)** Left panel: representative pictures showing c-Fos staining of spinal cord dorsal horn (SCDH) of sham and LAS model groups. Summary is shown on the right. **(B)** Left panel: representative pictures showing GFAP staining of SCDH of sham and LAS model groups. Summary is shown on the right. **(C)** Left panel: representative pictures showing CD68 staining of spinal cord dorsal horn (SCDH) of sham and LAS model groups. Summary is shown on the right. **(D)** Left panel: representative pictures showing p-ERK staining of SCDH of sham and LAS model groups. Summary is shown on the right. **p < 0.01, *p < 0.05. *n* = 5–6 mice/group. Student's *t*-test was used for statistics.

The spinal cord is vital for pain signal integration and relay to the higher brain regions (Gao and Ji, 2010; Hu et al., 2020). Therefore, we continued to examine the spinal neuronal and glial cell activities in LAS model mice. Immunostaining identified that the number of c-Fos positively labeled neurons was significantly increased in the ipsilateral spinal cord dorsal horn (SCDH) of LAS model mice compared with sham group mice on day 14 (Figure 6A). In addition, the immune reactivity for GFAP (a marker for astrocyte) and CD68 (a marker for spinal microglia) was significantly enhanced in ipsilateral SCDH of LAS model mice compared with sham group mice (Figures 6B, C). Moreover, we examined immunostaining of p-ERK, a molecule important for central sensitization and chronic pain (Gao and Ji, 2009). The immune reactivity for p-ERK was significantly increased in ipsilateral SCDH of LAS model mice vs. sham group mice (Figure 6D).

### Ibuprofen reduced pain in LAS model mice

**Figure 7:**
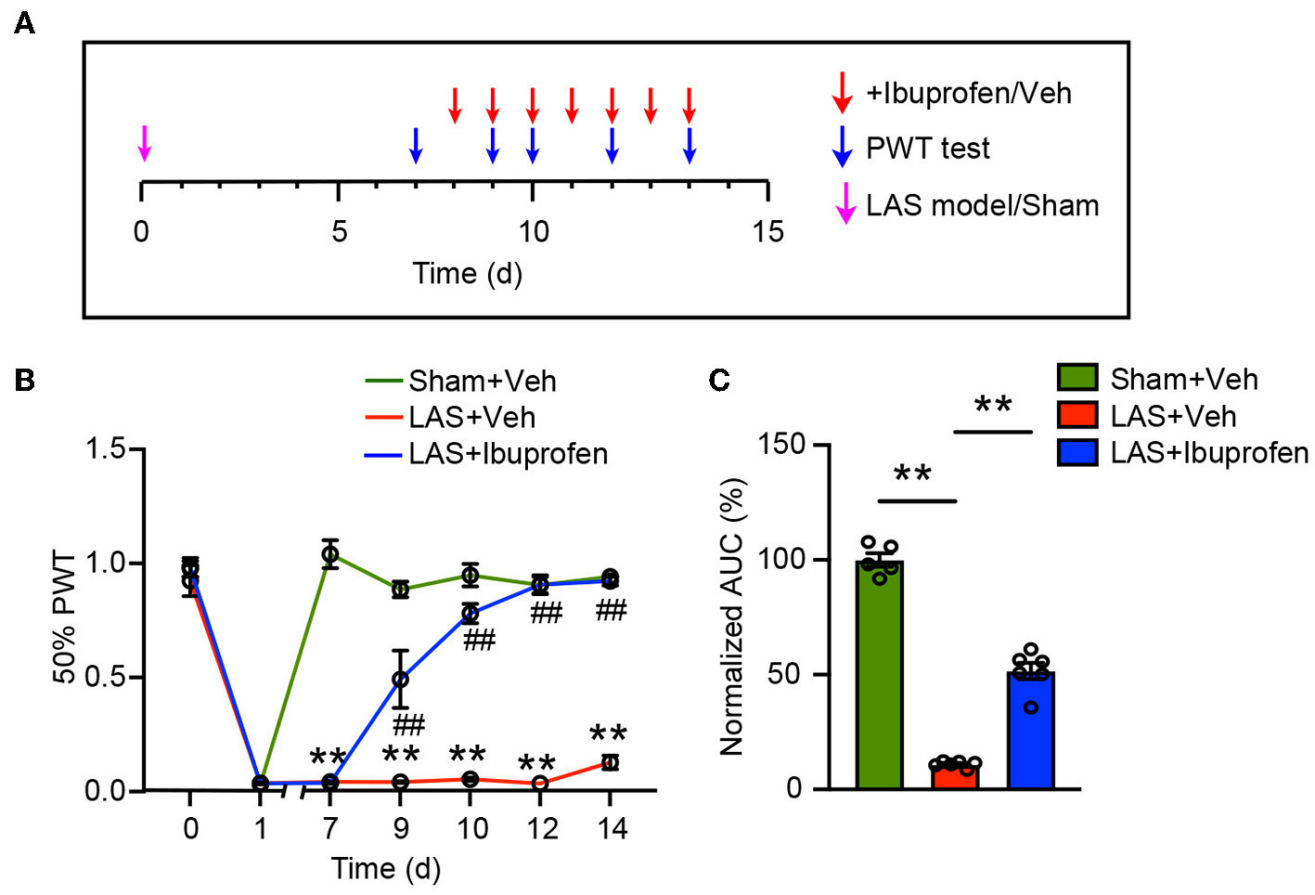
Mechanical pain in LAS model mice can be attenuated by ibuprofen. **(A)** Schedule for ibuprofen/vehicle treatment and PWT measurement. **(B)** Time course showing the effect of ibuprofen (30 mg/kg, i.p.) on mechanical pain hypersensitivity of LAS model mice. **p < 0.01 vs. Sham+Veh group. ^##^*p* < 0.01 vs. LAS + Veh group. **(C)** AUC analysis of curves in **(B)**. **p < 0.01. *n* = 5–6 mice/group. Two-way ANOVA followed by Tukey's *post-hoc* test was used for statistics in **(B)**. One-way ANOVA followed by Bonferroni's *post-hoc* test was used for statistics in **(C)**.

Non-steroidal anti-inflammatory drugs were commonly used to relieve ankle sprain-induced pain in human patients (Osborne and Rizzo, 2003). To further verify the translational significance of this model, we tested whether ibuprofen could alleviate pain-related behavior in LAS model mice. Ibuprofen (30 mg/kg, i.p.) or vehicle was administered to LAS model mice once daily starting from day 8 (Figure 7A). Ibuprofen treatment significantly inhibited mechanical hypersensitivity of LAS model mice (Figure 7B). AUC analysis shows the integrated effects of ibuprofen on LAS model mice (Figure 7C). This result indicates that the LAS model mice respond to drugs clinically used to treat ankle sprain pain.

## Discussion

Here, we established the LAS mouse model to mimic ankle sprain. We systematically evaluated pain-related behaviors in this mouse model. The LAS model mice developed obvious signs of mechanical and heat hypersensitivities as well as gait impairments in ipsilateral hind paws. LAS model mice also developed signs of pain-related emotional disorder, including aversion and anxiety. By RNA-Seq, we identified the DEGs and pathways that might contribute to the pain mechanisms of LAS model mice. In addition, LAS model mice also showed increased c-Fos and p-ERK immunoreactivity as well as glial cell overactivation in the ipsilateral SCDH. Finally, the LAS model mice respond to ibuprofen, a drug clinically used to treat ankle sprain pain. Therefore, we demonstrate that the LAS model mice showed persistent pain-related behavior that responds to clinically active drugs and may, therefore, be used to find novel targets or therapies for ankle sprain.

We found that LAS model mice presented with mechanical pain hypersensitivity that could last 1 week. This observation is consistent with one study showing that patients with acute ankle sprain showed signs of mechanical pain hypersensitivity (Ramiro-Gonzalez et al., 2012). In addition, we also observed heat pain hypersensitivity but not cold pain hypersensitivity in LAS model mice. Moreover, the LAS model mice also developed obvious signs of mechanical pain-related aversion behavior. NSAIDs are the most frequently used drugs clinically to relieve ankle pain in ankle sprain patients (Kosik et al., 2020). Here we found that ibuprofen can effectively attenuate mechanical pain hypersensitivity in LAS model mice. Therefore, our study provided a comprehensive evaluation of pain-related behavior in a mouse model of ankle sprain. These data indicate that this mouse model of ankle sprain may be further used for screening potential targets for treating ankle sprain-induced pain.

Emerging evidence indicate that sex-dimorphic mechanisms may mediate specific chronic pain condition (Chen et al., 2018). In clinical settings, females usually have a higher incidence of ankle sprains (Doherty et al., 2014). Therefore, in this study, we explored the pain-related behavior of female mice with ankles to see if a sex-dimorphic phenomenon may occur. Our study showed that female LAS model mice developed a similar degree of mechanical and heat pain hypersensitivities but no cold pain hypersensitivity after modeling. This result shows consistency with the results obtained in male mice. Thus, our results indicate that LAS model mice can develop mechanical and heat pain hypersensitivities, regardless of sex differences.

To investigate the potential mechanisms of pain in LAS model mice, we performed RNA-Seq of ipsilateral DRG of sham and LAS model mice groups. GO analysis found that the most enriched biological process includes immune response and positive regulation of gene expression. The immune response can trigger neuroinflammation, a critical process for peripheral sensitization, and contributes to chronic pain mechanisms. By RNA-Seq, we found several genes associated with the pain process that was upregulated in our study. *Mmp9* gene was among one of the DEGs we identified. Matrix metallopeptidase 9 (MMP9) is an enzyme in the zinc-metalloproteinases family that is involved in the degradation of the extracellular matrix (Ji et al., 2009). MMP9 is important for mediating neuroinflammation and participates in several neurological diseases including neuropathic pain (Kawasaki et al., 2008; Ji et al., 2009). It is reported that MMP9 is upregulated in the DRG of a mouse model of neuropathic pain. Moreover, MMP9 inhibition or genetic deletion attenuates neuropathic pain in the early phase. Mechanistic studies further reveal that MMP9 contributes to IL-1β activation in DRG, and MMP9 can be transported to central terminals in the spinal cord to activate microglia (Kawasaki et al., 2008). These findings imply a possible contribution of MMP9 in DRG to the pain mechanisms of LAS model mice by modulating pro-inflammatory cytokine production and activating spinal microglia. Future studies are needed to validate the protein expression as well as the activity of MMP9 in the DRG of LAS model mice.

In our study, the expression of *Mpo* is upregulated in ipsilateral DRG of LAS model mice. *Mpo* is a gene encoding myeloperoxidase, which is most abundantly expressed in neutrophil granulocytes and often used as a quantitative indicator for neutrophil activity (Rizo-Tellez et al., 2022). Neutrophil infiltration in DRG has been reported in experimental autoimmune encephalomyelitis (EAE) model mice and in chemotherapy-induced peripheral neuropathy (CIPN) model mice (Harada et al., 2019; Wang et al., 2023). Neutrophils may contribute to the mechanical allodynia of these model mice by releasing elastase, forming neutrophil extracellular traps (NETs), or producing excessive oxidative stress (Zhang et al., 2019; Yin et al., 2020b; Wang et al., 2023). These studies demonstrated the critical role of neutrophil infiltration in DRG in contributing to the pain mechanisms. Given the fact that neutrophil is considered an emerging type of cell participating in chronic pain, therefore, our findings suggest that neutrophil infiltration in DRG may be another possible mechanism underlying ankle sprain-induced pain. Further experiments will be needed to validate this observation from RNA-Seq.

*Pparg* encodes peroxisome proliferator-activated receptor-gamma isoform, which is a ligand-activated transcription factor belonging to a nuclear hormone receptor superfamily. PPAR ligands can produce anti-neuroinflammation activity by preventing the upregulation of inflammatory mediators (Maeda and Kishioka, 2009). Studies have shown that PPAR ligands can reduce animal models of inflammatory pain and neuropathic pain (Maeda and Kishioka, 2009). More specifically, PPARγ agonists can alleviate a variety of pain conditions, including trigeminal neuropathic pain, post-operative pain, and chemotherapy-induced peripheral neuropathic pain (Lyons et al., 2017; Santos et al., 2022; Zhang M. et al., 2022). In some studies, it has been found that PPARγ expression is decreased and contributes to pain mechanisms. However, in this study, we found that *Pparg* gene expression is significantly increased in ipsilateral DRG of LAS model mice. We are not clear on how to explain this contradiction. It could be possible that *Pparg* gene expression upregulation is an endogenous countermeasure against pain conditions in LAS model mice, which is reminiscent of endogenous opioids that are upregulated when pain occurs. Some similar findings were also observed in animal models of peripheral nerve injury in which the expression of PPARγ was elevated and may act to protect neurons from nerve injuries (Cao et al., 2012; Zhu et al., 2013).

Annexin-A1 (ANXA1) is a glucocorticoid-regulated protein that is responsible for the anti-inflammatory effects of glucocorticoids. Growing evidence indicates that ANXA1 can produce analgesic effects in a variety of animal pain models (Chen et al., 2014). ANXA1 may initiate antinociception through mechanisms such as the inhibition of pro-inflammatory cytokine production, the inhibition of neutrophil migration to the inflammatory site, and the interruption of pain signal generation by reducing TRPV1 activity (Chen et al., 2014; Zhang et al., 2021). In some studies, it has been reported that the *Anax1* gene expression was increased under certain pain conditions (Wang et al., 2009; Fineschi et al., 2022). Here, in this study, we found that the expression of the *Anxa1* gene that encodes ANXA1 is significantly upregulated in the DRG of LAS model mice. These findings suggest that ANXA1 may serve as an endogenous machinery for counteracting pain. Therefore, further studies are still needed to confirm the protein expression of PPARγ and ANXA1 in DRG of LAS model mice and explore their potential pathophysiological significance in ankle sprain-induced pain.

Chronic pain is usually accompanied by astrocytes and microglia overactivation in the SCDH (Ji et al., 2016). The overactivation of these spinal glial cells contributes to glial–neuron crosstalk, which can promote neuroinflammation and central pain sensitization (Ji et al., 2016, 2018, 2019). In our present study, we found that LAS model mice showed obvious overactivation of microglia and astrocytes in ipsilateral SCDH. Furthermore, c-Fos is activated in SCDH neurons, indicating persistent noxious stimulation coming from the periphery. We also observed that p-ERK expression is significantly upregulated in ipsilateral SCDH of LAS model mice. It is well-established that p-ERK induction in neurons of SCDH is a critical step for the development of central sensitization, and p-ERK in SCDH is often used as a reliable marker for central sensitization (Gao and Ji, 2009). Thus, our findings indicate that central sensitization occurs in ankle sprain model mice. This finding further indicates that central sensitization may happen in individuals with ankle sprain.

Overall, we have characterized in detail the pain-relative behaviors of a mouse model of the lateral ankle sprain. We further performed expression profiling of gene expression changes and analyzed the key signaling pathways by RNA-Seq in the primary sensory ganglia of the model mice. Certain DEGs and signaling pathways have been identified as possibly contributing to the pain mechanism of LAS model mice. Thus, our study suggests that the LAS mouse model can be used as a preclinical animal model for screening potential targets for treating pain associated with ankle sprain. Our study further provides insights into the mechanistic exploration of ankle sprain-induced pain.

## Data availability statement

The original contributions presented in the study are publicly available. The RNA-Seq dataset has been deposited into the National Center for Biotechnology Information's Gene Expression Omnibus repository with accession number GSE230178. This data can be found here: https://www.ncbi.nlm.nih.gov/geo/query/acc.cgi?acc=GSE230178.

## Ethics statement

The animal study was reviewed and approved by the Animal Ethics Committee of Zhejiang Chinese Medical University (Permission number: IACUC-20190819-04).

## Author contributions

YP, QH, YY, HN, CY, HW, and YT performed experiments and analyzed the data. QH, BoyuL, XH, and ZS participated in the model establishment. QH, JF, and BoyiL designed and supervised the study. YP and BoyiL wrote the manuscript. All authors reviewed the manuscript, read, and approved the final manuscript.
