# Supplementary material for: Characterization of pain-related behaviors and gene expression profiling of peripheral sensory ganglia in a mouse model of acute ankle sprain
Source: Front Behav Neurosci. 2023 May 25;17:1189489. doi: 10.3389/fnbeh.2023.1189489 (PMC10248128; doi:10.3389/fnbeh.2023.1189489)
Supplement: Supplementary file 1 [file Image_1.pdf]

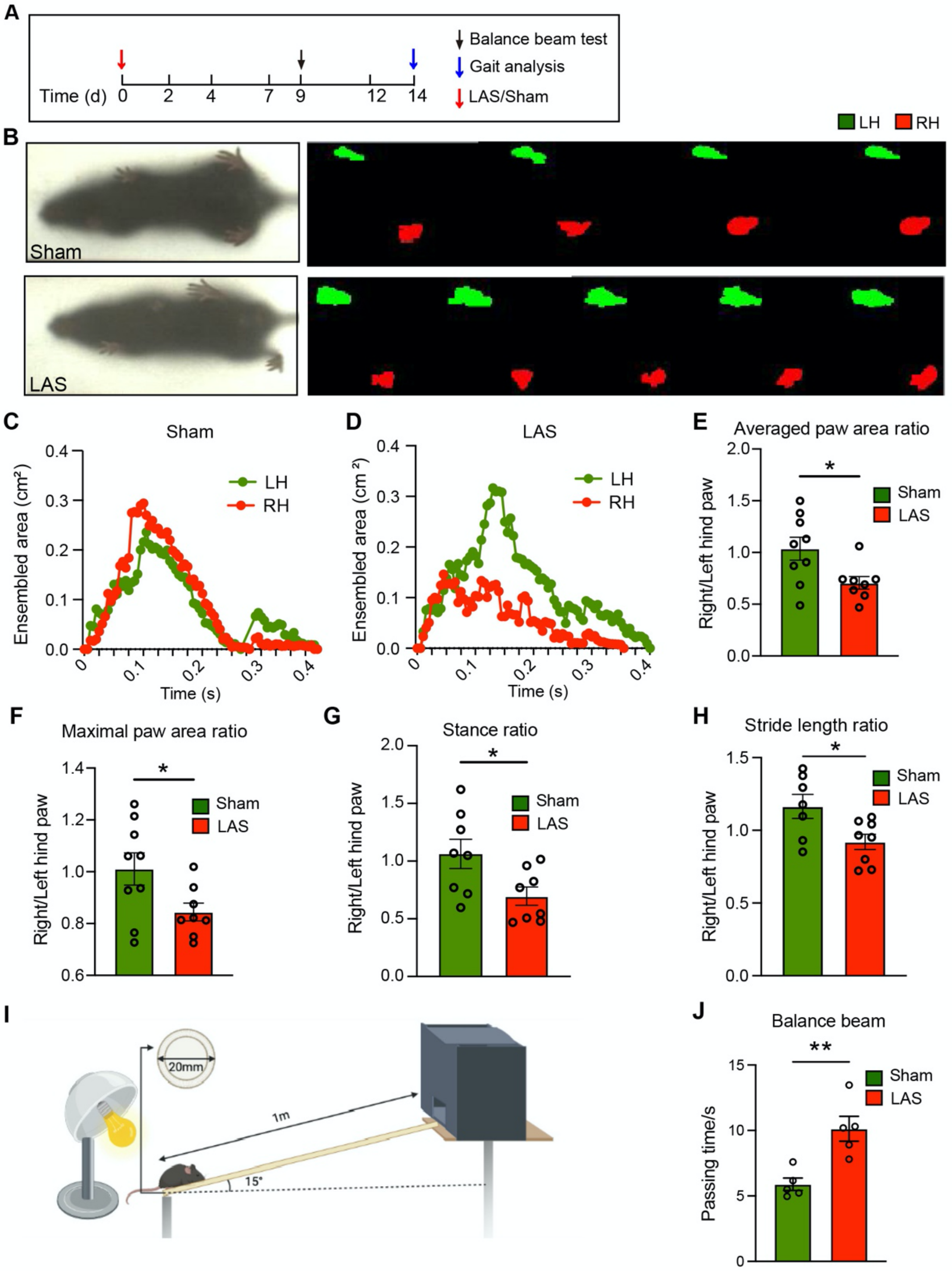

Suppl. Fig. 1 Gait and balance evaluation of LAS mouse model. (A) Time points for LAS model establishment, gait and balance analysis. (B) Representative pictures showing mice from sham and LAS model group recorded by gait analyzing system 14 days after model establishment. Left panel shows the bright field images of the mice on the treadmill. Right panel shows the series of hind paw prints being captured and analyzed. The letter LH or RH denote left or right hind paw respectively. (C&D)

Ensembled hind paw area of sham (C) and LAS model (D) mice. (E-H) Summary of averaged paw area ratio, maximal paw area ratio, stance ratio and stride length ratio of sham and LAS model mice. (I) Cartoon showing balance test of mice. (J) Summary for passing time from one end of the rod to the other side of sham and LAS model mice in the balance test. n=5-9 mice/group. $^{**}p<0.05$. Student's *t* test was used for statistics in panel E-J.
